# E-cigarettes and Vaping: A Smoking Cessation Method or Another Smoking Innovation?

**DOI:** 10.7759/cureus.32435

**Published:** 2022-12-12

**Authors:** Moteab Alotaybi, Saleh S Alzahrani, Abdulrahman M Algethmi, Nawaf S Alamri, Yaseen S Natto, Sara T Hashim, Abdulwahab Altammar, Afnan S Alzubaidi, Ibrahim B Alzahrani, Abdulkhaliq A Alghamdi

**Affiliations:** 1 Department of Primary Health Care, King Abdullah International Medical Research Centre, King Saud Bin Abdulaziz University for Health Sciences, Jeddah, SAU; 2 Department of Infectious Diseases, King Saud Medical City, Riyadh, SAU; 3 Medical Student, King Saud Bin Abdulaziz University for Health Sciences College of Medicine, Jeddah, SAU; 4 College of Medicine, King Saud Bin Abdulaziz University for Health Sciences, Jeddah, SAU; 5 Department of Forensic Medicine, Makkah Forensic Center, Makkah, SAU; 6 College of Medicine, King Saud Bin Abdulaziz University for Health Sciences, Riyadh, SAU; 7 Department of Family Medicine, Jaber Hospital, Kuwait, KWT; 8 Department of Emergency, Prince Meshari Bin Saud Hospital, Albaha, SAU; 9 College of Medicine, Albaha University, Albaha, SAU; 10 Rabigh College of Medicine, King Abdulaziz University, Jeddah, SAU

**Keywords:** cannabis, smoking cessation, smoking, vaping, e-cigarette

## Abstract

The prevalence of using e-cigarettes (vaping) has risen rapidly since its introduction in 2007, mostly among male youth. Although research on the health risks of e-cigarettes is still limited, there is growing evidence of debilitating pulmonary conditions and general immune weakness from e-cigarettes, leading to various infections. Moreover, there are concerns that vaping could be used as a new model of cannabis consumption, increasing cannabis addiction among adolescents. With well-known health risks from traditional smoking, e-cigarettes are viewed as a safe way of smoking, appealing more to youth. Additionally, extensive e-cigarette marketing boosted by the internet and fame has resulted in worries that e-cigarettes can lead to a renormalization of cigarette smoking and can be used as a new method to consume vaporized drugs. Although the concern that e-cigarettes are as harmful as traditional smoking has been raised, youth and most healthcare providers remain relatively unaware. Therefore, this review explored the association between e-cigarettes and traditional smoking. With the introduction of e-cigarettes in the last two decades, the topic is still new and less studied. Therefore, this review will help us understand the topic to better care for e-cigarette smokers and reduce the increasing public health burden from vaping.

## Introduction and background

The prevalence of using e-cigarettes has risen rapidly since the introduction of the product in the United States in 2007 [1,2]. The use of e-cigarettes in the last 30 days increased 10-fold from 1.5% in 2011 to 16% in 2015 among high school students in the United States, exceeding the prevalence of traditional cigarette smoking (9.3%) [2]. The e-cigarette is popular among both cigarette-smoking and never-cigarette-smoking youth [3-5].

E-cigarette smoking appears to vary by gender, with males more likely to smoke and prefer e-cigarettes over conventional cigarettes than females [6,7]. In Saudi Arabia, peer pressure and curiosity were reported to play a major role in e-cigarette use [8]. For Saudi smokers, having a smoking peer or family member was associated with smoking e-cigarettes [6]. E-cigarette use might be one of the contributors to nicotine addiction as e-cigarette users have a higher risk of becoming conventional cigarette smokers than non-users [9]. There are two designs for electronic cigarettes, namely, an open design featuring a main chamber refilled with e-liquid and a closed design that is reloadable or disposable with prefilled cartridges [10].

Traditional tobacco smoking has been used for centuries and studies have explored its harmful effects on health. Although e-cigarettes are considered a safer alternative to tobacco smoking, concerns have been raised about how e-cigarettes can renormalize cigarette smoking and become a new way for people to consume vaporized narcotics, especially young people. Therefore, this review aimed to explore the link between e-cigarette use and traditional smoking. This review will help comprehend the understanding of e-cigarettes to provide better care for e-cigarette smokers and reduce the growing public health burden associated with vaping and conventional smoking.

## Review

Relationship with traditional smoking

Concerns regarding the potential impacts of e-cigarettes on traditional cigarette smoking among young people have been rising with the popularity of e-cigarettes. The substances present in the smoke can likely make e-cigarettes more addictive, in addition to the nicotine delivery in e-cigarettes that has improved over time. This would indicate that their potential for addiction has grown, making them more appealing to smokers as an alternative to smoking and inciting non-smokers to start smoking [11]. However, the differences in levels of addiction between e-cigarettes and conventional cigarettes are not fully understood. Previous studies have identified a correlation between adolescent e-cigarette use and traditional cigarette smoking [12]. A systematic review and meta-analysis of longitudinal studies found strong and consistent evidence of an association between initial e-cigarette use and subsequent cigarette smoking initiation among adolescents and young adults aged 14-30 [12].

A study conducted in the United States among 8-10th graders in 2016 evaluating the relationship between e-cigarette use and conventional smoking found that the likelihood of ever smoking a traditional cigarette increased significantly with lifetime e-cigarette use by 2.49 times and with current e-cigarette usage by 2.32 times [13]. Similarly, another study from the United Kingdom conducted in 2016 found that people who had ever used an e-cigarette (53%) were more likely to start smoking than those who had never used an e-cigarette (8%) (odds ratio (OR) = 11.89, 95% confidence interval (CI) = 3.6-39.7). On the other hand, those who escalated their smoking (34%) versus those who did not (6%) were more likely to use e-cigarettes (OR = 5.79, 95% CI = 2.55-13.15). In the same study, those who were ever smokers (32%) versus never smokers (4%) had higher odds of smoking e-cigarettes (OR = 3.54, 95% CI = 1.68-7.45) [14].

The best evidence of a relationship between e-cigarette usage and traditional smoking from observational research was found to be smoking initiation. Although smoking initiation does not imply regular use, it can be viewed as a possible first step that can easily lead to regular use [11]. A systematic review of studies involving European and North American teenagers to determine if e-cigarette use was associated with the start of cigarette smoking in teenagers who had never smoked found that e-cigarette use was associated with the commencement of tobacco cigarette smoking. Teenagers who used e-cigarettes for 30 days were twice more likely to start conventional cigarette smoking [15]. Similarly, a study on Parisian adolescents aged 11-19 found that adolescents who solely used e-cigarettes were at higher risk of smoking tobacco than those who used other drugs or were non-smokers. Those whose best friends were traditional smokers were less likely to use e-cigarettes exclusively [14]. This aligns with another study that examined the decision influence of e-cigarette use to start smoking tobacco. The study concluded that e-cigarettes did not seem to be undermining the youth population’s long-term decline in traditional cigarette smoking in the United Kingdom [16].

A cohort study on Scottish adolescents who were followed up for one year found that 40.4% had used an e-cigarette at the baseline and 12.8% of the adolescents had never tried an e-cigarette at baseline. In line with the above-mentioned studies, after adjusting for having friends who smoke, family members’ smoking status, age, sex, family affluence, ethnic group, and school, those who had tried e-cigarettes at baseline had higher odds of traditional smoking (adjusted odds ratio (aOR) = 2.42, 95% CI = 1.63-3.60). This study found an interaction between e-cigarette use and smoking susceptibility as well as the use of e-cigarettes and smoking within the friendship group [17]. Another systematic review showed that adolescents were more likely to start using traditional cigarettes before switching to e-cigarettes. There is a higher possibility that they will start using the alternative product more frequently [18]. This indicates that the use of one product (traditional tobacco or e-cigarette) triggers the use of the other, especially among young people. Therefore, both the general public and healthcare professionals should be aware of the risks associated with both smoking methods. However, one research showed that most healthcare providers expressed uncertainties about the long-term dangers and safety of e-cigarettes [19].

E-cigarettes and vaping effects on health

E-cigarette use (often known as vaping) has a huge negative impact on the health of smokers. It consists of water with multiple chemicals, such as nicotine, glycerol, and propylene glycol, which are all transformed into vapor on inhalation [20]. Exposure to e-cigarettes weakens the innate immune system’s neutrophils, lung macrophages, and airway epithelium, leading to decreased epithelial barrier function and impaired phagocytosis. It also causes higher levels of inflammatory markers throughout the body and aberrant mucus composition caused by decreased mucociliary clearance and increased mucin and neutrophils [21,22].

It has been demonstrated that these innate immune deficiencies in the lung increase the pathogenicity of common respiratory pathogens, such as the influenza virus, *Staphylococcus aureus*, and *Streptococcus pneumoniae*, as well as the adhesion of several bacteria and fungi [23]. In case of immune deficiency, the adhesion of neutrophils to the endothelium, tissue matrix, and microbes is disrupted, and, therefore, immune cells are unable to emigrate to the sites of infection and fight them [24]. Unfortunately, most long-term health implications of vaping have not yet been identified and studied due to its relatively recent rise in popularity. However, preliminary studies indicate that vaping poses a few health and safety issues [25]. E-cigarette smoking was found to be associated with 2.1 times more obstructive lung function impairment [26], and computed tomography (CT) scans performed on e-cigarette smokers showed local inflammation with impaired gas exchange caused by aerosolized oils from e-cigarettes [27]. In addition, there have been reports of accidents and physical injuries from e-cigarette use. A total of 238 cases of e-cigarettes-related injuries were reported by 159 publications and the Centers for Disease Control and Prevention (CDC). More than half (53%) of the injuries were traumatic and caused by e-cigarette explosions or self-combustion, 24% by respiratory issues, and 12% by poisoning [28]. Cases of newborn problems, oral, cardiovascular, immunologic, hematologic, and allergic reactions have also been reported [28].

Conventional cigarette smoking is universally known to lead to poor dental health through local inflammation, debilitated innate immunity, and accelerated periodontal and gingival disease [29]. However, few studies have analyzed the impact of e-cigarettes on oral health. For instance, according to a study that involved 456,343 adults from across the United States, over half of the respondents (51.5%) reported having at least one permanent tooth removed in their lifetime because of tooth decay or gum disease [30]. Daily e-cigarette use was reported by 1.1% of respondents and was independently associated with 78% higher odds of poor oral health (aOR = 1.78, 95% CI = 1.39-2.30) [30]. Another systemic review showed increased plaque, marginal bone loss, clinical attachment loss, pocket depth, and reduced bleeding on probing among e-cigarette smokers [31].

The vapor induces toxicity, oxidative stress, mucus hypersecretion, inflammatory reaction, and hypersensitivity to the airway lining [32,33]. E-cigarette users inhale vapor created by heating a solution containing a psychoactive substance, most frequently nicotine or tetrahydrocannabinol (THC), as well as flavors and other additives. The pulmonary dangers of vaping are fast increasing, with the disease known as e-cigarette or vaping-associated lung injury (EVALI) and vaping product use-related lung harm being the most concerning [34]. EVALI is characterized by breathing difficulties, fever and chills, coughing, vomiting, diarrhea, headaches, dizziness, tachycardia, and chest pain and is clinically diagnosed. EVALI is treated symptomatically with a supportive approach [35]. In 2019, the CDC declared an outbreak after the death of several young people with lung injuries. Reports showed that EVALI symptoms or cases started to increase significantly in August 2019 and peaked in September 2019. As of February 18, 2020, 2,807 hospitalized EVALI cases or deaths from all 50 states, the District of Columbia, and two United States territories had been reported to the CDC, with 68 verified fatalities in 29 states and the District of Columbia [36].

Although smoking causes 11% of cardiovascular deaths globally, the effects of e-cigarettes on the cardiovascular system are yet to be clarified, even though e-cigarettes have been suggested to be less dangerous than conventional cigarettes due to their lower concentration of harmful substances. However, a recent study published in 2022 suggested that e-cigarette users have a higher risk of coronary heart disease, arrhythmia, chest discomfort, and other cardiovascular symptoms [37].

Cannabis use in association with e-cigarettes and vaping

There is an increase in the use of e-cigarettes as an easy mode of nicotine consumption among adolescents [38]. Therefore, there is a strong assumption that cannabis and other illicit drugs could spread among the youth to be consumed via vaping as one of the consumption methods, unlike the usual forms, such as edible forms, ingestible oil such as oleoresin, combustible smoking of cannabis, pills or capsules, topical creams, sprays, and patches [39]. However, a cohort study among 9,828 adolescents found a significant association between the use of e-cigarettes and the subsequent use of cannabis. In the same cohort, e-cigarette use was associated with a two times higher likelihood of cannabis use after one year [40]. In contrast, another study found that e‑cigarette users were less likely to use cannabis (aOR = 0.15, 95% CI = 0.09-0.25) [41]. A study involving 20,675 students showed that three times more e-cigarette users (33.3%) used cannabis than all students (12.4%) [42]. Another study reported that the use of e-cigarettes was linked to nearly a four-fold increase in the likelihood of starting and regularly using cannabis [1]. Interestingly, an online survey reported that most (61%) cannabis users also used vaping as a method, and 12% preferred vaping over any other method for the consumption of cannabis [43].

Prevention of vaping

More than 100,000 people die due to smoking each year in the United Kingdom, making it the primary preventable cause of sickness and early mortality [44]. About 47.5 million persons use tobacco; nearly 70% wish to stop, and 42.5% make an annual attempt. To increase abstinence rates, smoking cessation programs should combine medication with behavioral and/or cognitive counseling to be successful [45,46]. To encourage patients to stop smoking, doctors might use the five As framework (ask, advise, assess, assist, and arrange). Every time a patient sees a doctor, they should be questioned about their tobacco use and evaluated for motivation to give it up. If patients are not yet ready to stop smoking, doctors should employ motivational interviewing approaches and firmly counsel them to do so [47].

There have been programs to raise awareness and prevent vaping among youth, such as the Youth Vaping Prevention Program targeting 5-12th graders using the Catch My Breath curriculum designed to prevent the start of e-cigarette use among adolescents and developed by the Michael & Susan Dell Center for Healthy Living at the University of Houston School of Public Health in the United States [48]. The program teaches about the dangers of vaping, its effects on the body, how to identify false marketing, and how to resist peer pressure and provides vaping cessation counseling [49].

The American Lung Association Vape-Free Schools Initiative aims to support students affected by e-cigarettes; offer guidance, education, and cessation toolkits; and involve students, parents, school staff, and the community to prevent e-cigarette smoking among young people [50].

## Conclusions

The prevalence of using e-cigarettes has risen rapidly since their introduction, and concerns regarding the potential impacts of e-cigarettes on health have been growing. Studies have indicated that e-cigarettes have similar health consequences as traditional tobacco use, including weakening immunity, infections, dental issues, and increased risk of cardiovascular diseases. There is a positive correlation between adolescent e-cigarette use and strong and consistent evidence of an association between initial e-cigarette use and subsequent cigarette smoking initiation, mainly among adolescents and young adults. Therefore, e-cigarettes should be considered as harmful as traditional tobacco smoking, and preventive measures should be adopted in addition to raising awareness campaigns to educate the general public, youth, and healthcare professionals about this relatively new type of smoking with a relatively poor understanding of its impacts.
